# Optical Investigation of Individual Red Blood Cells for Determining Cell Count and Cellular Hemoglobin Concentration in a Microfluidic Channel

**DOI:** 10.3390/mi12040358

**Published:** 2021-03-26

**Authors:** Ann-Kathrin Reichenwallner, Esma Vurmaz, Kristina Battis, Laura Handl, Helin Üstün, Tivadar Mach, Gabriele Hörnig, Jan Lipfert, Lukas Richter

**Affiliations:** 1 Technologies for Precision Medicine, Siemens Healthcare GmbH, Günther-Scharowsky-Str. 1, 91058 Erlangen, Germany; annkathrin.reichenwallner@gmx.de (A.-K.R.); esmavurmaz96@outlook.de (E.V.); kristinabattis@t-online.de (K.B.); laura.handl@fau.de (L.H.); helin.uestuen@outlook.de (H.Ü.); tivadar.mach@siemens-healthineers.com (T.M.); 2 Department of Physics and Center for Nanoscience, LMU Munich, Amalienstr. 54, 80799 Munich, Germany; jan.lipfert@lmu.de; 3 Product Lifecycle Management, Siemens Healthcare GmbH, Röntgenstr. 19-21, 95478 Kemnath, Germany; gabriele.hoernig@siemens-healthineers.com

**Keywords:** blood analysis, RBC counting, cellular hemoglobin concentration, cellular volume, microfluidics, hydrodynamic focusing, digital holographic microscopy

## Abstract

We demonstrate a blood analysis routine by observing red blood cells through light and digital holographic microscopy in a microfluidic channel. With this setup a determination of red blood cell (RBC) concentration, the mean corpuscular volume (MCV), and corpuscular hemoglobin concentration mean (CHCM) is feasible. Cell count variations in between measurements differed by 2.47% with a deviation of $-0.26\times 10^{6}$ μL to the reference value obtained from the Siemens ADVIA 2120i. Measured MCV values varied by 2.25% and CHCM values by 3.78% compared to the reference ADVIA measurement. Our results suggest that the combination of optical analysis with microfluidics handling provides a promising new approach to red blood cell counts.

## 1. Introduction

A routine quantitative blood analysis known as a complete blood count (CBC) includes the enumeration of cellular populations, leukocyte differentiation, and the determination of hematocrit, cellular hemoglobin (HB), and mean corpuscular volume (MCV) of red blood cells (RBC) [1,2,3,4]. The CBC is used for diagnosing hematologic diseases for monitoring a patient’s medical condition or the process of treatments over time, and for assessing overall health [5].

Nowadays, automated hematology analyzers use high-throughput techniques for cell counting, such as impedance, flow cytometry, and light scatter, which allow for a performance of up to 120 samples per hour [6,7]. Large statistical information is provided by these systems, but to achieve the goal of high throughput, manufacturers use makeshift methods to acquire fast and accurate results. For example, abnormal cases are flagged for further manual review, which is in general performed under a basic light microscope [8]. Another example is the fast measurement of cellular hemoglobin concentration, which is obtained by lysing RBCs and the resulting solution is photometrically investigated by using a known wavelength [1,9,10]. Here, the obtained HB concentration is a mean value over a known number of RBCs and is referred to as the mean cellular hemoglobin concentration (MCHC) [11]. Contrary to most analyzers, Siemens ADVIA 2120i additionally provides an RBC cytogram, where the volumes and hemoglobin concentrations of individual RBCs are visualized. In the ADVIA system, isovolumetrically sphered RBCs pass through a beam of monochromatic light, which produces a forward light scattering pattern. At two selected angular intervals (low angle (2${}^{\circ }$–3${}^{\circ }$) and high angle (5${}^{\circ }$–15${}^{\circ }$)), the individual scatter signals are converted into volume and refractive index values based on the Mie theory of light scattering on homogeneous spheres [6,12,13]. By taking the mean value of the RBC hemoglobin concentration histogram, the resulting parameter is the corpuscular hemoglobin concentration mean (CHCM), which is the mean HB concentration of individual RBCs [6,11,14]. An analysis of the RBC cytogram pattern allows the diagnosis of e. g. iron deficiency anemia, thalassemia, dual deficiency anemia [15], or a thrombocytopenia-associated multiple organ failure [16].

In the current study, we present two fluidic methods, which allow the determination of RBC concentration in a microfluidic channel by counting cells during a known period and calculating the corresponding blood volume. In the first fluidic approach the number of cells is approximated by time and channel width, while the second method uses hydrodynamic focusing of the sample stream which allows for the counting of all cells during a selected period. In the same microfluidic channel, we optically investigate individual sphered cells to calculate their cellular hemoglobin concentration and volume by basic light microscopy and digital holographic microscopy (DHM). To verify our results, we compare our measurements of individual cells to CHCM results obtained from Siemens ADIVA 2120i, as the CHCM represents the mean HB concentration of individual RBCs.

## 2. Materials and Methods

### 2.1. Samples

Peripheral human blood was drawn with informed consent and procedures approved by application 316_14B of the ethical commission of the Friedrich-Alexander University Erlangen-Nürnberg, Germany, from healthy donors and collected in 4.7 mL ethylenediaminetetraacetic acid (EDTA)-coated tubes preventing blood coagulation. Clinical samples were obtained from the Department of Medicine 5—Hematology and Oncology, Erlangen, Germany. All samples were processed within 4 h of collection. Isovolumetric sphering of erythrocytes was achieved by diluting whole blood 1:630 in ADVIA 2120/2120i RBC/PLT (Siemens Healthineers, Erlangen, Germany) reagent containing sodium dodecyl sulphate (SDS) and glutaraldehyde [17]. We obtained reference values of cell count and hemoglobin content for each donor from performing a complete blood count on a Siemens ADVIA 2120i. A centrifugation step (5 min at 400 g) and subsequent uptake in phosphate buffered saline (PBS, Miltenyi Biotec, Bergisch Gladbach, Germany) was necessary to obtain a final blood concentration of 1:300. Sample fixation and reference measurements on the ADVIA 2120i were performed within one hour to ensure sample stability [18].

### 2.2. Microscopic Technologies

**Figure 1:**
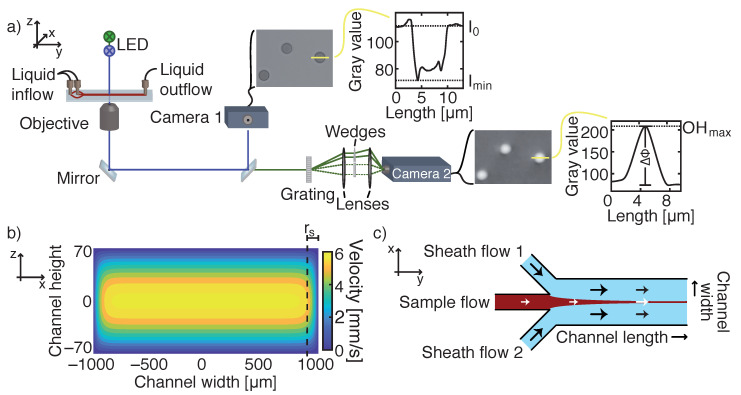
(**a**) Microscopic setup equipped with two LEDs emitting light in blue and green. The light beam travels through a microfluidic channel, where blood cells absorb the light. A dichroic mirror splits the blue light beam, which is captured by a charged-coupled device (CCD)-sensor (camera 1), from the green beam. A diffraction grating is placed in the green path, which separates the sample beam from a reference wave field. By recombining both diffracted beams, a phase shift proportional to a cell’s refractive index and thickness can be observed by a second camera. The yellow line depicts the height profiles of either transmission or phase images. (**b**) The velocity flow profile of a laminar flow in a rectangular microfluidic channel for a flow rate of Q = 0.06 mL/min. The side region r${}_{s}$ indicates the part of the flow profile where the fluid velocity at the channel mid-height deviates by more than 2.5% from the maximum velocity. Rectangular cross section is not to scale. (**c**) Illustration of hydrodynamic focusing of a sample flow by two adjoining sheath flows. By tuning the flow rates of each inlet, the width of the sample stream can be adjusted to the FOV (field of view) of the camera.

Images of blood cells were acquired by combining two optical technologies, basic light microscopy and digital holographic microscopy (DHM) in a single microscopic configuration (Figure 1a). A custom-built microscope purchased from Ovizio Imaging Systems, Belgium, combines two light beams from two independent light sources. Light emitting diodes (LEDs, Dragon1 PowerStar Colors, Osram, Thatcham, UK) were selected to either emit blue light at 455 nm or green light at 530 nm. The light paths of the simultaneously triggered LEDs are combined via a dichroic mirror and travel through a microfluidic channel. After the sample plane, a 40× achromatic objective (numerical aperture (NA) = 0.75, Nikon) magnifies the sample plane. Another dichroic mirror splits the light beam again into two separate paths. The blue path is captured directly by a monochromatic CCD-sensor (Grasshopper3 USB3, FLIR) (Figure 1a, camera 1), while the green light beam enters a patented [19,20] differential digital holographic module. In this module a diffraction grating, placed at the input plane, generates different diffraction orders, where the zero-order diffraction is referred to as the reference wave, while the first order diffraction carries the phase information of the sample. The phase content of an investigated object depends on variations in the refractive index and physical height. Wedges can adjust path differences between the two waves, while filters can select the desired diffraction orders. Both wave fronts are recombined under a certain angle at the camera plane (Grasshopper3 USB3, FLIR) (Figure 1a, camera 2). This single-shot technique can acquire fast (100 fps, acquisition time 20 μs) and stable images with nanometer resolution [21].

A second setup, which only incorporated basic light microscope technology, used another blue LED (λ = 425 nm). Further optical components were identical to the previous combined setup. The 425 nm—LED was used for additional cell concentration measurements. Our camera field of view (FOV) captured an area of the microfluidic channel of 176 μm × 132 μm.

### 2.3. Microfluidics

#### 2.3.1. Microfluidics—Setup Configurations

Our microfluidic glass chip was manufactured by IMT Masken und Teilungen AG, Greifensee, Switzerland, by applying wet-chemical etching of a glass slide with hydrofluoric acid [22,23]. A thin # 1.5 coverslip sealed the microfluidic channel with its rounded edges. To simplify theoretical flow profile calculations, we assumed a rectangular cross-section of the microfluidic channel as the aspect ratio α≪1. The channel design comprised of three inlets having identical dimensions in width and height (h = 144 μm), which merge into a 2000 μm wide and 1.56 cm long channel, and a single outlet reservoir. A neMESYS Base120 pump system with three modules (CETONI GmbH, Korbussen, Germany) was equipped with 10 mL gas tight syringes (VWR, Darmstadt, Germany) and used to control flow velocity and hydrodynamic focusing of cells [24].

#### 2.3.2. Microfluidic Flow Profiles

Two experimental fluidic approaches, a single-inlet and a triple-inlet pressure driven configuration, were realized for cell concentration measurements. For both configurations the glass chip contained three inlets, while in the single-inlet configuration, sheath flow inlets 1 and 2 (Figure 1c) were blocked. In the first method, blood cells enter through the center inlet and distribute homogeneously along the full channel width. Only a proportion of cells is recorded in the observed FOV. The flow rate was set to 0.06 mL/min. The theoretical flow profile is given by a series solution of the Navier–Stokes equation. A Poiseuille parabolic profile can be used as an approximated solution in rectangular channels with small aspect ratios (h/w → 0), meaning a laminar flow profile can be assumed [25,26,27,28,29]. Maximum and mean fluid velocities are calculated from the corresponding velocity field (Figure 1b). The single inlet method is applicable for counting RBCs and platelets, where the distribution of cells along the channel width does not significantly increase the measurement time by detecting a sufficient number of rare blood cells, such as basophils.

The second fluidic approach, symmetric hydrodynamic focusing, squeezes a sample flow in between two sheath flows (autoMACS Rinsing Solution, Milteny Biotec, Bergisch Glasbach, Germany (PBS, 2 × 10${}^{3}$ M EDTA, pH 7.2)) [24,30,31,32,33,34], which induce a two-dimensional enrichment of blood cells along the channel width (Figure 1c). The channel geometry comprised of three inlets with different flow rates for sheath ($Q_{\mathrm{sheath}}=0.11\;\mathrm{mL}/\min)$ and sample flow ($Q_{i}=0.017\;\mathrm{mL}/\min)$. The width of the hydrodynamically focused stream w${}_{f}$ can be predicted, if the flow rate of the sample sheath Q${}_{i}$, the average velocity of the focused stream ${\bar{\nu }}_{f}$, and the height of the channel h are known [30]:

(1) $$w_{f}=\frac{Q_{i}}{{\bar{\nu }}_{f}\cdot h},$$

where ${\bar{\nu }}_{f}=8.49\;$mm/s was calculated from the laminar flow profile, where we assumed a laminar flow profile with equal fluid velocities for sheath and sample flow in the region around the FOV. The theoretical focused stream width for Q${}_{i}=0.017\;$mL/min was w${}_{f}=231.88\;$μm. Although w${}_{f}$ is larger than the width of our FOV, the majority of the floating cells was pushed into one FOV. Vertical focusing of cells was achieved by a method that underlies a company secret. In this method, almost every cell during a certain time period is observed and recorded. Therefore, it would be possible to count leukocytes with the triple inlet method. To obtain enough data during our cell concentration measurements, we solely used RBCs, as less RBCs get lost during the dilution and sphering process than during the leukocyte purification process.

### 2.4. Experiments

A LabVIEW (National Instruments, Austin, USA)-based program purchased from Fraunhofer Institute for Microengineering and Microsystems IMM, Mainz, Germany, triggered the pumps and controlled flow rates of the glass syringes for flow establishing. A washing step prior to the experiment was necessary to remove cell fragments from previous experiments. Afterwards, cells were pushed into the microfluidic channel and image recording started with a frame rate of 100 fps for cell concentration measurements using the laminar flow approach and for cellular HB and volume experiments and 20 fps for cell concentration measurements using hydrodynamic focusing. Exposure time was set to 20 μs for laminar flow measurements, 11 μs for hydrodynamic focusing measurements, and 5 μs for cellular HB and volume experiments for both transmission and phase images. The number of recorded images was 1000 for laminar flow experiments, 4000 for hydrodynamic focusing experiments, and 3000 for determining cellular HB and volume. After each experiment, we performed two washing steps, first by using 2.5% sodium hypochlorite and second by using 0.1 N hydrochloric acid to destroy the remaining blood cells and proteins. To neutralize the acid a rinsing step with PBS was performed in between measurements. In between measurements, the microfluidic channel was continuously filled with liquids and one liquid was directly replaced with another.

### 2.5. Image Processing and Statistical Analysis

Image processing was performed by an in house-built JavaScript program. Transmission images and phase images have different methods for background correction. Background correction was necessary to homogenize the overall intensity of recorded images. For transmission images, background correction was achieved by subtracting a single background image taken of a cell-free channel from each image. In phase images, the phase shift values for each pixel were converted to grey values. Grey scale images are further used for subtracting a background image, where the pixel by pixel median grey value from the first 11 images of an experiment was subtracted from all grey scale phase images.

Then, a binary mask was generated based on a fixed segmentation threshold of the grey scale images. The detection of an object’s contour was performed on the binary masks. Additional parameters based on the pixel values inside an object’s contour and based on the co-occurrence matrix [35] were calculated. These parameters are summarized in a table, in addition with the image and cell identification number.

The table was loaded into a MATLAB (MathWorks Inc., Natick, USA) script, which counted detected cells, filtered in-focus cells, and calculated cellular HB concentration (c${}_{\mathrm{HB}}$) and cellular volume of individual cells. Furthermore, the mean values of cell concentration, c${}_{\mathrm{HB}}$, and cellular volume were taken for individual donors of up to 10 measurements per donor and compared to the CHCM and MCV obtained from Siemens ADIVA 2120i, respectively. The determination of absolute mean differences between our measurements and the reference system was generated by fitting a linear line with slope 1 to our data points and extracting the intercept of the *y*-axis. The distribution of data points around the fit was referred to as measurement variations.

The filtering of detected objects, which were used for cell concentration analysis, was performed on transmission images and was solely based on the value of cell area, which had to be larger than 9.5 μm${}^{2}$, to remove platelets and artifacts. The distinction of several individual cells inside a cell cluster was based on differentiating clouds in a diameter vs. cell area plot (Supplementary Figure S1). No further selection of cells being in- or out-of-focus was used, as the aim was to count all passing cells.

Filtering of cells, which were used for calculating cellular HB concentration and cellular volume, excluded out-of-focus cells, platelets, and leukocytes and other occurring artifacts. In transmission images, a cell’s parameters had to comply with the following filters: Aspect ratio < 1.1, cell area > 5 μm${}^{2}$, circularity > 0.83, energy > 0.15, mass center shift < 1.25, optical height minimum < 62, and radius variance < 1. For phase images, the following filters were applied: Aspect ratio < 1.1, cell area < 40 μm${}^{2}$, circularity > 0.85, dissimilarity > 3.2, radius variance < 0.5, and sphericity > 0.95. Parameter explanation can be found in Supplementary Table S2.

For transmission and phase images, two ways of calculating the cellular HB concentration exist. The hemoglobin absorbance contributes most to attenuation in transmission images, yielding dark cells on a bright background. Calculating the cellular hemoglobin concentration c${}_{\mathrm{HB}}$ in transmission images is achieved by applying the Beer–Lambert law [3]

(2) $$c_{\mathrm{HB}}=\frac{-\log\left(\frac{I}{I_{0}}\right)\cdot M}{\varepsilon d},$$

where I, I${}_{0}$ are transmitted and background intensity, respectively, molar mass M, molar attenuation coefficient ε, and diameter *d* of the observed sample. As a RBC consists of 96% of hemoglobin, it is reasonable to use the values for hemoglobin ($M_{\mathrm{HB}}=64458\;g/\mathrm{mol}$, $\varepsilon_{\mathrm{HB}}=116933\;\mu m^{-1}/\left(\mathrm{mol}/\mathrm{mL}\right)$ at a wavelength λ=425nm) and neglect the absorbance occurring from the cell’s membrane [36]. The value of the HB molar attenuation coefficient is the sum of a gaussian profile that is added to the wavelength dependency of the molar attenuation coefficient for oxygenated HB. The mean of the gaussian profile equals the LED wavelength and the standard deviation corresponds to the bandwidth of the LED, which was estimated to be 10 nm [37] (Supplementary Figure S2). The cellular absorbance can be measured in two ways: Taking the mean intensity (I${}_{\mathrm{mean}}$) over the whole cell area or by taking the minimum intensity (I${}_{\min}$) inside the cell’s contour. Extracting both intensities and the diameter of an individual cell from the transmission mask, enabled us to calculate the cellular hemoglobin concentration for each individual cell.

In DHM images, the phase content, which can be expressed by the optical height (OH) variance ΔΦ of individual cells, can be extracted from hologram images. The optical height variance relates to the refractive index Δn and the thickness d of the sample:

(3) $$\Delta \Phi =\frac{2\pi }{\lambda }\cdot \Delta n\cdot d,$$

where λ is the wavelength and $\Delta n=n_{s}-n_{m}$ is the difference of the refractive index of the sample and the surrounding medium [3,38,39]. However, the refractive index of a RBC depends on its hemoglobin concentration, resulting in $n_{s}=n_{\mathrm{HB},0}+\beta c_{\mathrm{HB}}$, with $n_{\mathrm{HB},0}=1.337$ being the effective refractive index of a RBC at zero HB concentration and β=0.1497mL/g the refractive increment at 530 nm [2]. Using $n_{m}=n_{\mathrm{PBS}}=1.336$, the HB concentration in an individual cell can be calculated by:

(4) $$c_{\mathrm{HB}}=\frac{1}{\beta }\left(\frac{\Delta \Phi \cdot \lambda }{2\pi \cdot d}-n_{\mathrm{HB},0}+n_{m}\right).$$

For phase value extraction three possibilities exist: Taking the mean optical height (OH${}_{\mathrm{mean}}$) value over the whole cell area, the maximum optical height (OH${}_{\max}$) value inside a cell’s contour, or the optical volume (OV), which is the integration of the phase value inside the segmented contour [40]. Extracting the phase difference and the diameter of a cell from the recorded images allowed for the calculation of cellular volume and HB concentration.

Therefore, for calculating hemoglobin concentration and volume we used the parameters diameter, intensity inside the cell’s contour, and phase shift inside the cells contour for transmission or phase images, respectively.

## 3. Results

### 3.1. Cell Concentration

A determination of cell concentration is feasible if the cell count can be attributed to a known volume. Normal values of the RBC count per μL are $4.6-6.2\times 10^{6}$ for adult males and $4.2-5.4\times 10^{6}$ for adult females [10]. For our two fluidic approaches, we derived formulas for calculating cell concentration, where the cell count depends on the dilution of the investigated sample, on the number of recorded objects in one FOV compared to the real number of cells present in the whole microfluidic channel, on cell migration velocities, and the corresponding blood volume.

#### 3.1.1. Laminar Flow Approach

**Figure 2:**
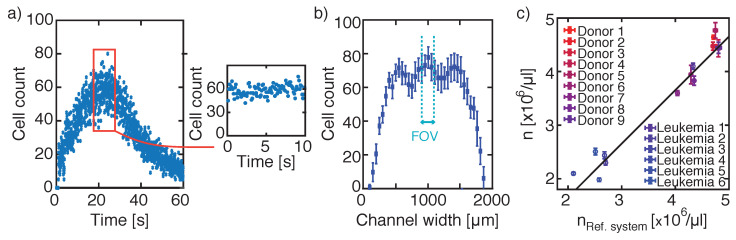
Laminar flow approach for cell counting. (**a**) Cell count per frame over 60 s, where during a selected time period of 10 s the cell count per frame is constant (red square and inlet). (**b**) Cell distribution along the channel width, where the dashed lines indicate the centre FOV for further cell concentration measurements. (**c**) Comparison of cell concentrations obtained from different donors measured with the reference system Siemens ADVIA 2120i to cell concentrations resulting from the laminar flow approach. To calculate the actual number of cells and the corresponding volume out of cells passing the FOV in a certain time period, time, channel width, and flow velocity are extrapolated. Variations in measurements were 2.47 ± 1.46% with an absolute deviation to the reference system of $-0.26\times 10^{6}/$μL.

After entering the microfluidic channel through a single inlet, blood cells were evenly spread across the channel width. Due to the large packing density of RBCs, we studied a blood solution diluted by a factor of 300. After starting the pressure driven flow, we recorded all passing cells in one FOV for a period of 60 s (Figure 2a) and identified a time region of 10 s, where the cell count per frame is constant over time (Figure 2a, inlet). In this allocated period, no dilution effects happened in the direction of the flow during the fluid transportation process, which we verified with a high concentration of new methylene blue solution (data no shown), and therefore we assumed a stable RBC dilution D of 1:300. After the shown 60 s, the majority of the sample load has passed and the cells are further diluted by the successive PBS-solution. The number of recorded cells during the constant 10 s was between 20,000 and 25,000 cells, depending on the absolute cell concentration. The following measurements were recorded in this time frame.

The width of our FOV is much smaller than the channel width, which required an extrapolation of the number of observed cells with the real number of cells passing the cross section of the microfluidic channel. We determined the fraction of observed cells in the center FOV by moving the FOV 0.2 mm/s perpendicularly to the flow direction within 10 s along the entire channel width. The resulting cell count along the channel width is shown in Figure 2b, where each data point represents the mean cell count per frame with error bars for 25 consecutive frames. The width of the center FOV is indicated by the light blue dashed lines. Blood cells do not spread homogeneously across the whole channel width. There is a small region close to the channel wall where no cells at all were detected. By further analyzing the width of the cell sheath, we found w${}_{\mathrm{sheath}}=1801\pm 13\;$m. To calculate the number of FOVs, to detect all passing cells, we obtained an image multiplication factor of:

(5) $$I_{\mathrm{cells}}=\frac{w_{\mathrm{sheath}}}{w_{\mathrm{FOV}}}=10.2.$$

As cells were not homogeneously distributed along the channel width, we had to introduce a cell count distribution factor δ, which homogenizes the location of detected cells. By dividing the mean number of detected cells in one frame by the maximum number of cells per frame in the center region, we determined the distribution factor to be δ=0.79±0.02.

The last important feature for calculating cell count was the determination of cell migration in between two consecutive images. As all cell velocities were based on a laminar flow profile, we knew theoretically the velocity of every cell. However, the width of one FOV did not cover the whole channel width and the migration distance of every cell was approximated by dividing the velocity field (Figure 1b) into two sections: A side region r${}_{s}$, consisting of velocities deviating more than 2.5% of the maximum velocity and a center region r${}_{c}$, comprising of velocities deviating less than 2.5% of the maximum velocity. The center region is highlighted in yellow in Figure 1b and is located with r${}_{s}=162.15$ μm from the channel side walls.

Next, we calculated the presence of an individual cell in the very same frame. Therefore, we determined the mean migration distance ${\bar{d}}_{c,s}$ of RBCs in consecutive images by multiplying the mean section velocities (${\bar{u}}_{s}$ = 4.691 mm/s, ${\bar{u}}_{c}$ = 5.606 mm/s) with the time difference Δt = 1/frame rate (frame rate = 100 Hz) between two images. The theoretical value $\tau_{c,s}$ of how often we record an individual cell in consecutive images becomes:

(6) $$\tau_{c,s}=\frac{l_{\mathrm{FOV}}}{{\bar{d}}_{c,s}},$$

which resulted in $\tau_{c}=2.35$ and $\tau_{s}=2.81$. This means that an individual cell is captured 2.35 times in the center region and 2.81 times in the side region. As we now know how far cells travel depending on their position along the channel width, we had to correct the absolute number of recorded cells by the percentage of cells traveling through the center or side region:

(7) $$\frac{w_{\mathrm{channel}}-2r_{s}}{w_{\mathrm{channel}}}\;\mathrm{and}\;\frac{2r_{s}}{w_{\mathrm{channel}}},$$

respectively.

To calculate a RBC concentration per μL of blood, we had to determine the corresponding fluid volume, which passes the microfluidic channel during our recording time. Therefore, we computed the observed volume of a FOV compared to the channel volume in considering the length of a FOV, multiplied by the number of recorded images and by a fluid velocity correction factor. The fluid velocity correction factor adjusts the velocity of the fast-moving cells in the center region to the mean velocity of the fluid ${\bar{u}}_{\mathrm{fluid}}=3.556$ mm/s. The observed volume V becomes:

(8) $$V=A_{\mathrm{cs}}\cdot l_{\mathrm{FOV}}\cdot \frac{\#\mathrm{frames}}{I_{\mathrm{cells}}}\cdot \frac{{\bar{u}}_{c}}{{\bar{u}}_{\mathrm{fluid}}},$$

with A${}_{\mathrm{cs}}$ being the cross-section of the microfluidic channel. The observed blood fluid volume for 10 s is 5.72 μL out of the loaded 35 μL inside the channel. Changing the valve positions due to pre-measurement preparations resulted in a volume loss.

The resulting equation for calculating cell concentration n [cells/μL] in a laminar flow profile with a single-inlet microfluidic channel geometry then gets:

(9) $$n=\frac{D\cdot I_{\mathrm{cells}}\cdot \delta \cdot \left(\frac{\#\mathrm{cells}}{\tau_{c}}\cdot \frac{w_{\mathrm{channel}}-2r_{s}}{w_{\mathrm{channel}}}+\frac{\#\mathrm{cells}}{\tau_{s}}\cdot \frac{2r_{s}}{w_{\mathrm{channel}}}\right)}{V}.$$

Finally, we verified our measured RBC count of nine healthy donors and six leukemia patients with the mean values obtained from the reference system Siemens ADIVA 2120i and found a linear correlation of our results with variations to the fit of 2.47±1.46%. The y-intercept of the linear slope was $-0.26\times 10^{6}/$μL (Figure 2c). Even for abnormal cell counts found in leukemia patients, the measured RBC concentration shows the same trend compared to values obtained from the reference system.

Although the single-inlet laminar flow approach uses simplifications, the cell concentration is determined in agreement to the reference system.

#### 3.1.2. Hydrodynamic Focusing Approach

**Figure 3:**
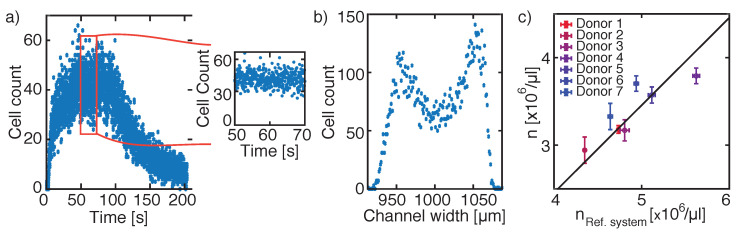
Hydrodynamic focusing approach. (**a**) Cell counting per frame over 210 s, where during a selected time period of 20 s the cell count is constant (red square). (**b**) Cell distribution along the centre part of the channel width. As all cells are recorded in the centre FOV during the dedicated 20 s, solely a volume determination is necessary for determining the corresponding cell concentration. (**c**) Comparison of cell concentrations obtained from different donors measured with the reference system Siemens ADVIA 2120i to cell concentrations resulting from applying the hydrodynamic focusing approach. A linear correlation is found for the measured seven healthy donors with variations to the solid line of 2.22 ± 1.74%. However, the y-intercept for the straight line was located at $-1.62\times 10^{6}/$μL.

In our hydrodynamic focusing set-up geometry, the flow rates of the sample and the sheath flows were tuned to adjust the width of the sample stream to the width of the FOV. By moving the FOV along the channel width, we observed that the duration of the sheath formation takes approximately 50 s, followed by 20 s of constant cell count per frame. Figure 3a shows the time dependency of the cell count per frame in the channel center and the cell count during the constant 20 s is highlighted. The following measurements were performed during a period where the cell count per frame was constant over time.

The sheath width for hydrodynamically focused RBCs for a sample flow rate Q${}_{i}$ of 0.017 mL/min was found to be less than the width of a FOV (Figure 3b), in contrast to the theoretical calculated sheath width (w${}_{f}$ = 231.88 m) [30]. We assumed that the RBCs should be distributed along the theoretical sheath width and multiplied the obtained cell count by a fraction of w${}_{f}$/w${}_{\mathrm{FOV}}$.

Additionally, we had to calculate the distance an individual cell travels in between two consecutive images. The mean velocity of the fluid for a total flow rate of:

(10) $$Q_{\mathrm{tot}}=Q_{i}+Q_{\mathrm{sheath}}=0.127\;\mathrm{mL}/\min$$

becomes ${\bar{u}}_{\mathrm{fluid}}$ = 8.486 mm/s with a maximum velocity in the center region of ${\bar{u}}_{c}$ = 12.729 mm/s. Using Equation (6) with Δt = 50 ms, we found $\tau_{c}$ = 0.21, which means, that we captured every 5th cell. During the constant 20 s, we recorded 400 images and captured between 18,000–25,000 cells for a dilution factor of D = 150. However, increasing the frame rate to 100 fps, which corresponded to Δt = 10 ms, would be enough to capture every passing cell. For our experiments, we did not increase the frame rate due to the large amount of generated data, however the setup would allow a faster recording time.

Taking everything together, we obtained a cell concentration formula by applying symmetric hydrodynamic focusing:

(11) $$n=\frac{D\cdot \frac{w_{f}}{w_{\mathrm{FOV}}}\cdot \frac{\#\mathrm{cells}}{\tau_{c}}}{V},$$

with V = Q${}_{i}\cdot \#$ frames·Δt. The observed blood fluid volume for 20 s is 5.67 L.

To verify our cell concentration formula used for hydrodynamic focusing experiments, we measured the blood of seven healthy donors. The mean value of several runs of an individual sample was compared to the corresponding mean value obtained from the reference system, Siemens ADIVA 2120i. We obtained a linear correlation of our results with variations to the fit (Figure 3c, solid line) of 2.22 ± 1.74%. Although we found a linear correlation between our measurements and the reference system, the y-intercept of the linear fit differed by $-1.62\times 10^{6}/$μL (Figure 3c).

To summarize the part for determining cell concentration in a microfluidic channel, we obtained more similar results to the reference values by using the single-inlet laminar flow approach than for the hydrodynamic focusing approach.

### 3.2. Cellular Volume and Hemoglobin Concentration

Two additional important erythrocyte parameters in a complete blood count are the computation of cellular volume and the calculation of cellular hemoglobin concentration. First, we investigated the erythrocyte volume and hemoglobin concentration in a microfluidic channel by a basic transmission light microscopy approach. Normal values of the mean corpuscular volume (MCV) are 76–100 fl and for the cellular hemoglobin concentration mean (CHCM) 29.0–34.0 g/d [6]. For a total number of optically detected cells of 20,000–25,000, approx. 90% were detected as single cells and 6000–13,000 cells ($\hat{=}$ 30–50%) were used for calculating cellular volume and HB concentration.

**Figure 4:**
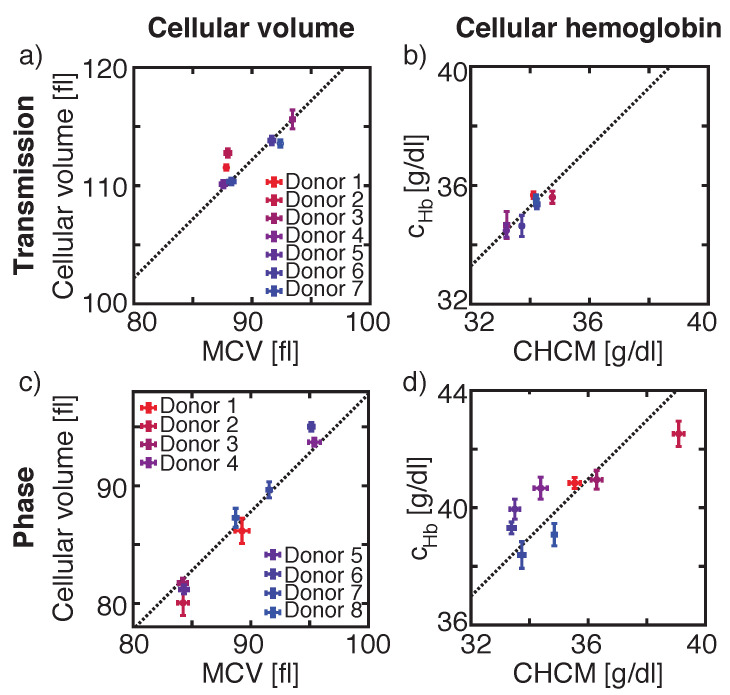
Comparisons of the cellular volume of our measurements to the reference value mean corpuscular volume (MCV) obtained from ADVIA 2120i for transmission (**a**) and phase images (**c**) and of the microscopic measured cellular hemoglobin concentration to the reference value corpuscular hemoglobin concentration mean (CHCM) obtained from ADVIA 2120i for transmission (I${}_{\min}$) (**b**) and phase images (OH${}_{\mathrm{mean}}$) (**d**). Same set of donor samples were used for V and Hb measurements in transmission images and another set of donors was used for V and Hb values calculated from phase images.

The cellular volume of sphered erythrocytes is obtained by calculating the volume of a sphere, where the RBC’s radius is extracted from the recorded images. Comparing the mean of the measured cellular volume of sphered RBC of seven healthy donors to the MCV obtained from the reference system, we found a correlation between both parameters with variations to the linear fit with slope 1 of 0.65% (Figure 4a, dashed line). However, the y-intercept of the fit varied by 22.2 fl to the absolute value obtained from the reference system, resulting in a deviation of 25.05%.

The same donors, which were used for determining cellular volume in transmission images, were used for calculating cellular HB concentration by either using the mean intensity I${}_{\mathrm{mean}}$ value over the whole cell area, or by using the minimum intensity I${}_{\min}$ value inside a cell’s contour. A comparison of the mean of the measured cellular HB, obtained by using I${}_{\mathrm{mean}}$ to ADVIA’s CHCM, showed a linear dependency between both values (Figure 4b). Here, the mean values varied by 0.43% from the linear fit and the y-intercept of the fit was 1.28 g/dL corresponding to 3.87% (Figure 4b, dashed line). By using I${}_{\min}$, the mean difference between the reference system and the measurement was −2.65 g/dL, corresponding to 7.75%, and the measurement variations towards the fit were 0.55% (data not shown).

The segmentation of a cell in transmission images strongly depends on the vertical position of the recorded cell inside the microfluidic channel, as the strength and number of diffraction rings surrounding the cell influences the segmentation of the cell and hence the determination of its area (Supplementary Figure S3). The calculated cellular volume does not reflect the actual volume. Therefore, we studied another optical approach, DHM, where the depth of focus (DOF) of our DHM setup configuration [41] was ± 1.26 μm and is intrinsically larger than the DOF of the light microscopy setup [42] (± 0.49 μm). Hence, the DHM is less sensitive to the vertical position of a cell and smaller variations occur.

Studying another eight different healthy donors for phase images, resulted in variations of the mean of the measured cellular volume towards the linear fit with slope 1 by 0.77% (Figure 4c, dashed line). The y-intercept of the fit deviates by −2.2 fl or 2.25% from the ADVIA value.

Same donors were used to calculate cellular HB concentration extracted from phase images. The linear fit with slope 1 of the mean cellular HB concentration values obtained from OH${}_{\mathrm{mean}}$ varies by 4.98 g/dL or 14.36% from ADVIA’s CHCM value (Figure 4d) with inter-measurement deviations of 1.75% towards the fit. Similar values were calculated for OH${}_{\max}$ with a y-intercept of the fit of 6.40 g/dL, corresponding to 17.91% and inter-measurement deviations of 1.94% (data not shown). A larger deviation between the experimental and reference value was found for calculating the HB concentration by using the OV. Here, inter-measurement variances were 1.67% with a y-intercept of the linear fit of 9.84 g/dL, corresponding to a difference of 27.79% to the reference CHCM value (data not shown). A summary of all values obtained for transmission and phase images in comparison with the reference system are summarized in Table 1.

A unique feature of the Siemens ADVIA 2120i is the determination of cellular volume and cellular HB concentration on a cell-by-cell basis. Both parameters are visualized in a RBC cytogram, which enables a fast identification of e. g. iron deficiency anaemia [15], and allow a calculation of the red blood cell volume distribution width (RDW) and the hemoglobin distribution width (HDW) based on individual cells. The RDW reflects the degree of heterogeneity of erythrocyte volume, which is increased in several diseases, such as cardiovascular disease, venous thromboembolism, cancer, and diabetes [43,44,45]. The HDW determination is important for the early detection of iron deficiency anaemia [46].

**Figure 5:**
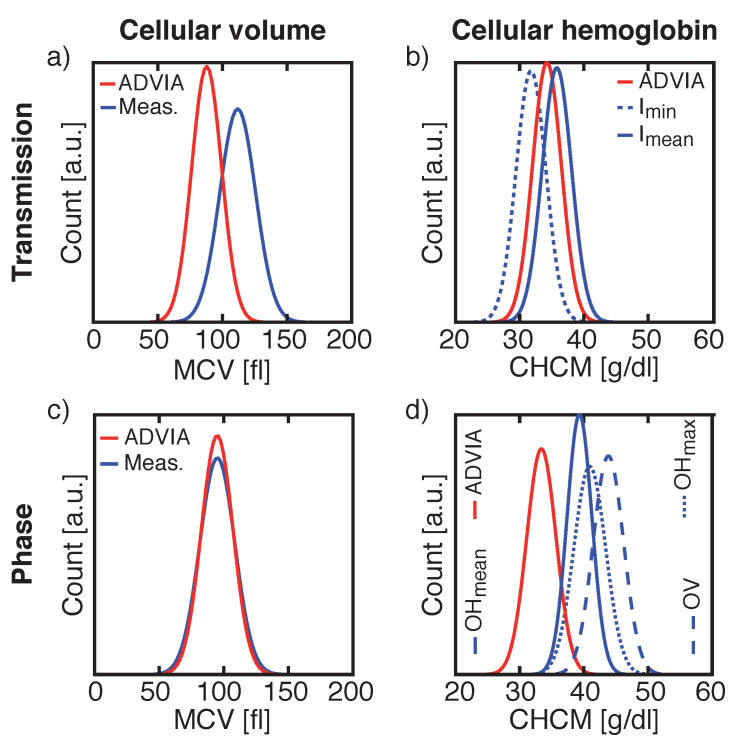
Comparisons of the cellular volume distribution widths and the absolute mean cellular volume (MCV) values of the reference system and our measurements for transmission (**a**) and phase images (**c**) and of the cellular hemoglobin distribution widths with its absolute mean cellular hemoglobin concentration (CHCM) values of the reference system and our measurements for transmission (**b**) and phase images (**d**). Same donor was used for V and Hb measurements in transmission images and another donor was used for V and Hb values calculated from phase images.

Cellular volume and hemoglobin distribution widths and the absolute values of the MCV and CHCM were compared between our measured results and data obtained from the ADVIA 2120i for both transmission and phase images for two individual samples (Figure 5). In the RBC cytogram, both parameters are combined into a single plot.

**Figure 6:**
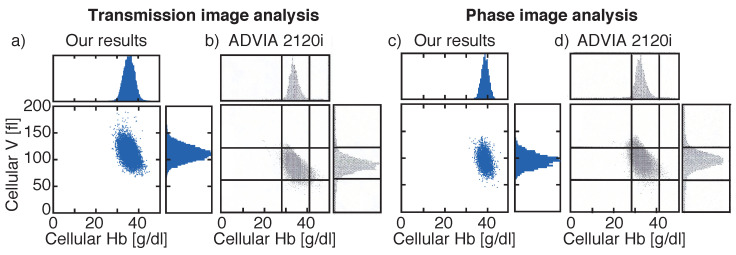
Comparison of red blood cell (RBC) cytograms showing cellular volume and cellular hemoglobin obtained by our systems for transmission (**a**) or phase images (**c**) and obtained by the reference system Siemens ADVIA 2120i (**b**,**d**). Different blood samples were investigated for transmission and phase images. Histograms highlight the distribution widths. Vertical lines in (**b**,**d**) are located at 28 g/dL and 41 g/dL and horizontal lines at 60 fl and 120 fl. Vertical axes are all the same for all plots.

RBC cytograms calculated from transmission (Figure 6a or phase images Figure 6c) recorded by our system are compared to cytograms obtained from Siemens ADVIA 2120i for the same donors (Figure 6b,d). Here, the same Gaussian shaped distribution widths were found for both measuring cellular volume and HB concentration for transmission and phase images (Table 2), but with the off-sets of the mean values already mentioned in Figure 4 and Table 1.

## 4. Discussion

Determining the cell concentration in our microfluidic channel by using approximations of time and channel width resulted in concordant values found for healthy donors and leukemia patients. Although we used approximations for the laminar flow approach, the overall error seemed to be sufficiently small as the mean variations of the experimental values to the reference system were $-0.25\times 10^{6}/$μL. Variations in the measurements occurred as the fluidic workflow was not perfectly stable over several days and had to be recalibrated. However, we achieved reasonable inter-measurement variations of 2.47%.

Although, the inter-measurement variations for cell concentration measurements based on symmetric hydrodynamic focusing were slightly smaller with 2.22%, the y-intercept of the linear fit of the mean values was positioned at $-1.62\times 10^{6}/$μL.

Detecting all cells would allow the counting of leukocytes, as cell enrichment was achieved by the symmetric hydrodynamic focusing, if RBCs were lysed or removed prior to the measurement process. Overall, good results were achieved by using the laminar flow approach. Further measurements should be performed with a more stable system for counting cells based on hydrodynamic focusing.

Volume determination was equally accurate for intensity and phase images with measurement variations towards the fit by less than 0.77%. However, the values for the absolute cellular volume derived from phase images differed less compared to values obtained from transmission images (4% vs. 25%). In transmission images, the number and size of diffraction rings varied based on the vertical position of the cell, which led to wrongly segmented cells and additionally contributed to the cellular volume. This is shown by the large variation of approx. 25% between our results calculated from transmission images and MCV values obtained from Siemens ADVIA 2120i. Based on the contribution of the diffraction pattern to the cellular volume, larger cells became even larger, which results in ΔRDW${}_{\mathrm{Transmission}}$ = 0.68 fl, which is the difference of RDW obtained for our intensity measurements and ADVIA 2120i, compared to ΔRDW${}_{\mathrm{Phase}}$ = 0.45 fl for phase images. Hence, our results for optically determining cellular volume obtained from phase images were closer to the ADVIA MCV than for transmission images. Reproducibility in between measurements was comparable for transmission and phase images.

In contrast, the calculation of the cellular hemoglobin concentration based on Beer–Lambert’s law achieves more reproducible results (<0.55%) than the calculation based on phase differences (<1.67%). Additionally, the absolute values obtained from transmission images differed by approx. 4% to the reference values compared to at least 14% for phase images. Furthermore, the difference between the HDW calculated by Siemens ADVIA 2120i and our results for transmission images was smaller than for phase images (ΔHDW${}_{\mathrm{Transmission}}<\Delta$HDW${}_{\mathrm{Phase}}$). Yet, for both images analyses, best results were obtained either for I${}_{\mathrm{mean}}$ or OH${}_{\mathrm{mean}}$. Occurring defective pixels might cause larger variations for HB calculation based either on I${}_{\min}$ or OH${}_{\max}$ and OV. For I${}_{\mathrm{mean}}$ and OH${}_{\mathrm{mean}}$ such variations are counterbalanced.

An advantage of our presented prototype compared to the Siemens ADVIA 2120i is that we capture images of blood cells, which can further be used for an additional reanalysis of a certain blood sample to achieve a detailed diagnosis, e.g., in detecting malaria in RBCs. In the Siemens ADVIA 2120i, only indirect side scatter signals produce the HB and CHCM values. Furthermore, less blood volume is needed in our measurements, as we have less tubing and less processed dead volume. Therefore, the overall biological hazardous waste is smaller in our prototype, than in the Siemens ADVIA 2120i.

Although we experienced differences for our calculated mean HB and V values compared to the mean values obtained from the reference system, the shape of the distributions for RDW and HDW were in good agreement to the reference system. This might result from the different filter parameters both systems used to identify analyzable cells.

## 5. Conclusions

We showed that the determination of RBC concentration in a microfluidic channel is possible by using two different approaches, either by evenly distributing cells over the whole channel width and applying a laminar flow approach or by hydrodynamically focusing the sample stream to fit into a FOV. In the first approach, where only a fraction of the cells was recorded, several approximations were necessary to determine the corresponding volume, whereas in the second method the majority of cells was recorded. As the fluidic system was not perfectly stable due to blood cell engorgement in the connection tubes between syringe and glass chip, we achieved better results for the laminar flow approach with its approximations.

In the second part of this article we presented our results on optically determining cellular volume and cellular hemoglobin concentration for chemically sphered RBCs. By using light microscopy or digital holographic microscopy, either the total amount of HB or the actual size of individual cells was calculated in agreement, with minor variations, to values obtained from the reference system Siemens ADVIA 2120i.

As a future perspective, it is interesting to calculate the volume of a biconcave RBC in its natural shape. By combining both equations used for determining cellular HB concentration in transmission and DHM mode, a volume calculation could be performed.

With the ambition to design a prototype that costs less, runs with smaller volumes, and has smaller dimensions than current bench-top systems we are able to distinguish different cell types label-free [24] and to determine cellular volume and hemoglobin concentration.

## Figures and Tables

**Table 1:** Deviations of MCV and CHCM values obtained by our measurements and by the reference system Siemens ADVIA 2120i.

	Transmission Images	Phase Images
**ΔMCV**	25.05% ± 0.65%	2.25% ± 0.77%
**ΔCHCM**	I${}_{\mathrm{mean}}$: 3.87% ± 0.43%	OH${}_{\mathrm{mean}}$: 14.36% ± 1.75%
	I${}_{\min}$: 7.75% ± 0.55%	OH${}_{\max}$: 17.91% ± 1.94%
		OV: 27.79% ± 1.67%

**Table 2:** Cellular volume distribution width (RDW) and cellular hemoglobin distribution width (HDW) comparisons of the reference system Siemens ADVIA 2120i and our measurements for two example samples. Comparison of the same sample between Siemens ADVIA 2120i and intensity measurements and another sample for comparing the reference method and our phase measurements.

	Transmission Images		Phase Images	
	ADVIA	Measurements	ADVIA	Measurements
**RDW**	13.20 ± 0.07 fl	13.88 ± 0.21 fl	12.93 ± 0.08 fl	13.32 ± 0.29 fl
**HDW**	2.22 ± 0.02 g/dL	I${}_{\min}$: 2.31 ± 0.04 g/dL	2.30 ± 0.01 g/dL	OH${}_{\max}$: 2.50 ± 0.08 g/dL
		I${}_{\mathrm{mean}}$: 2.26 ± 0.04 g/dL		OH${}_{\mathrm{mean}}$: 1.90 ± 0.09 g/dL
				OV: 2.22 ± 0.09 g/dL

## Supplementary Materials

The following are available online at https://www.mdpi.com/article/10.3390/mi12040358/s1.
