# Supplementary material for: Optical Investigation of Individual Red Blood Cells for Determining Cell Count and Cellular Hemoglobin Concentration in a Microfluidic Channel
Source: Micromachines (Basel). 2021 Mar 26;12(4):358. doi: 10.3390/mi12040358 (PMC8066749; doi:10.3390/mi12040358)
Supplement: Supplementary file 1 [file micromachines-12-00358-s001.zip › Supplement/micromachines-1145174-proofreading_AR_Supplement.pdf]

Supplementary Materials: Optical investigation of individual red blood cells for determining cell count and cellular hemoglobin concentration in a microfluidic channel

Ann-Kathrin Reichenwallner, Esma Vurmaz, Kristina Battis, Laura Handl, Helin Üstün, Tivadar Mach, Gabriele Hörnig, Jan Lipfert and Lukas Richter *

Supplementary Materials: The following are available online at https://www.mdpi.com/2072-666X/1/1/0/s1, Figure S1: Illustration of the distinction of individual cells inside a cell cluster for cell concentration measurements, Table S1: Explanation of filter parameters, Figure S2: Molar extinction coefficient for HB for the used LED system. Figure S3: Segmentation of a cell for different focal positions.

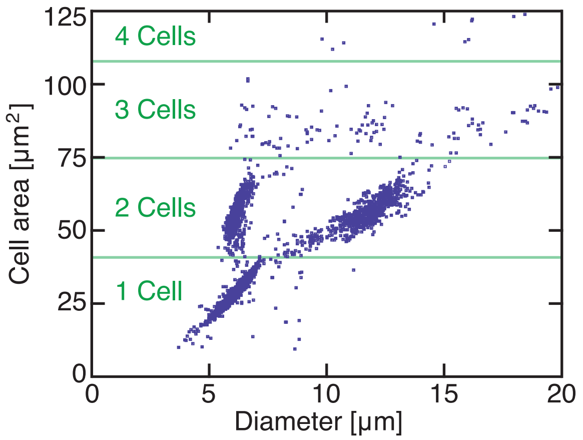

Figure S1. The distinction of several individual cells inside a cell cluster is based on differentiating clouds in a diameter vs. cell area plot. The cell area differences between the smaller and next larger cell clusters were 35 μm^2.

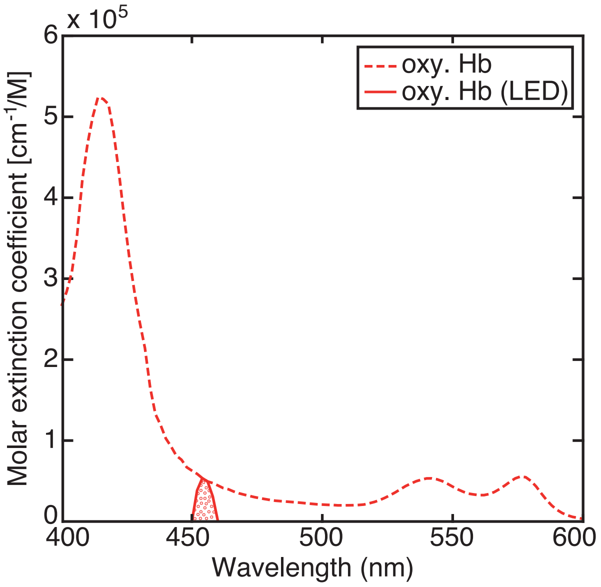

Figure S2. Molar extinction coefficient for HB for the used LED system. The dotted line indicates the molar extinction coefficient for oxygenated hemoglobin. The solid line the estimated hemoglobin absorbance of a red blood cell illuminated with a 425 nm - LED with an estimated bandwidth of 14 nm.

Table S0. Explanation of filter parameters.

Parameter	Unit	Explanation	Setup configuration
Aspect ratio	a.u.	max (width, height) / min (width, height)	Transmission, Phase
Cell area	μm^2	Area contained by cell contour	Transmission, Phase
Circularity	(0 – 1) a.u.	Circularity of the cell contour is calculated by 4π·cell area/perimeter^2. Circularity of a circle is 1.	Transmission, Phase
Dissimilarity	a.u.	Dissimilarity measure based on gray-level cooccurrence matrix (GLCM) of phase values converted to 6 bit grayscale image for the pixels inside cell contour	Phase
Energy	a.u.	Energy measure (sum of squared elements) based on GLCM of phase values converted to 6 bit grayscale image for the pixels inside cell contour.	Transmission
Mass center shift	a.u.	Euclidian distance between geometric centroid and mass centroid. Mass centroid is calculated out of the squared phase values.	Transmission
Optical height minimum (OH_{min})	a.u.	The minimum grey value inside the contour.	Transmission
Radius variance	a.u.	The variance of the distance between the centroid of the contour and each contour support point.	Transmission, Phase
Sphericity	$(-1-1)$ a.u.	A measurement for a spherical shape of the cell. Describes the correlation of phase values on a horizontal and vertical cut in the middle of the cell contour to an idealized spherical cell modeled by $-x^2+1$ is calculated.	Phase

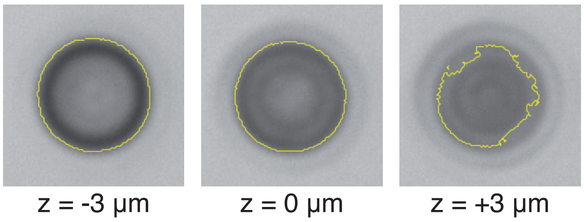

Figure S3. Segmentation of the same cell for three different vertical positions. Depending on the z-height of the cell, the calculated parameters differ. For example, for the cell area A: for z = - 3μm: A = 24.67 μm^2, for z = 0 μm: A = 23.57 μm^2, and for z = + 3 μm: A = 17.12 μm^2.
