# Supplementary figures and images for: Optical Investigation of Individual Red Blood Cells for Determining Cell Count and Cellular Hemoglobin Concentration in a Microfluidic Channel
Source: Micromachines (Basel). 2021 Mar 26;12(4):358. doi: 10.3390/mi12040358 (PMC8066749; doi:10.3390/mi12040358)

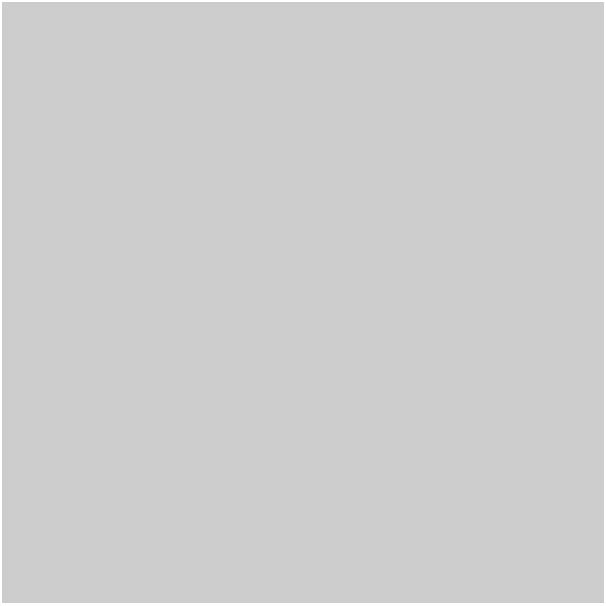

Supplement: Supplementary file 1 [file micromachines-12-00358-s001.zip › Supplement/Definitions/author2.jpg]

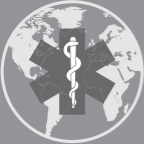

International Journal of  
*Environmental Research  
and Public Health*

Supplement: Supplementary file 1 [file micromachines-12-00358-s001.zip › Supplement/Definitions/ijerph-logo-eps-converted-to.pdf]

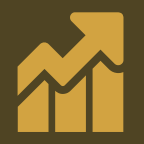

International Journal of  
*Financial Studies*

Supplement: Supplementary file 1 [file micromachines-12-00358-s001.zip › Supplement/Definitions/ijfs-logo-eps-converted-to.pdf]

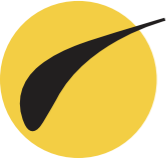

International Journal of

*Turbomachinery*

*Propulsion and Power*

Supplement: Supplementary file 1 [file micromachines-12-00358-s001.zip › Supplement/Definitions/ijtpp-logo-eps-converted-to.pdf]

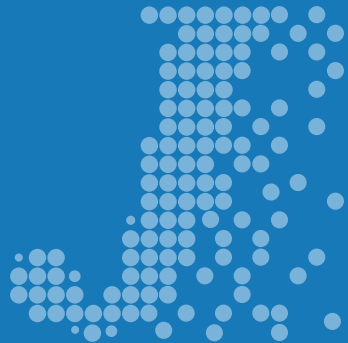

*J* Multidisciplinary  
Scientific Journal

Supplement: Supplementary file 1 [file micromachines-12-00358-s001.zip › Supplement/Definitions/j-logo-eps-converted-to.pdf]

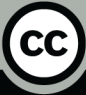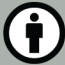

BY

Supplement: Supplementary file 1 [file micromachines-12-00358-s001.zip › Supplement/Definitions/logo-ccby-eps-converted-to.pdf]

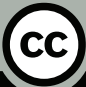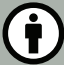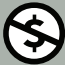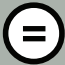

BY

NC

ND

Supplement: Supplementary file 1 [file micromachines-12-00358-s001.zip › Supplement/Definitions/logo-ccby-nc-nd-eps-converted-to.pdf]

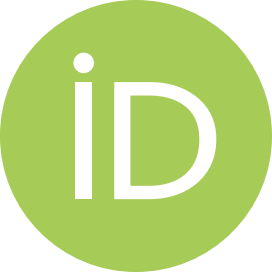

Supplement: Supplementary file 1 [file micromachines-12-00358-s001.zip › Supplement/Definitions/logo-orcid-eps-converted-to.pdf]

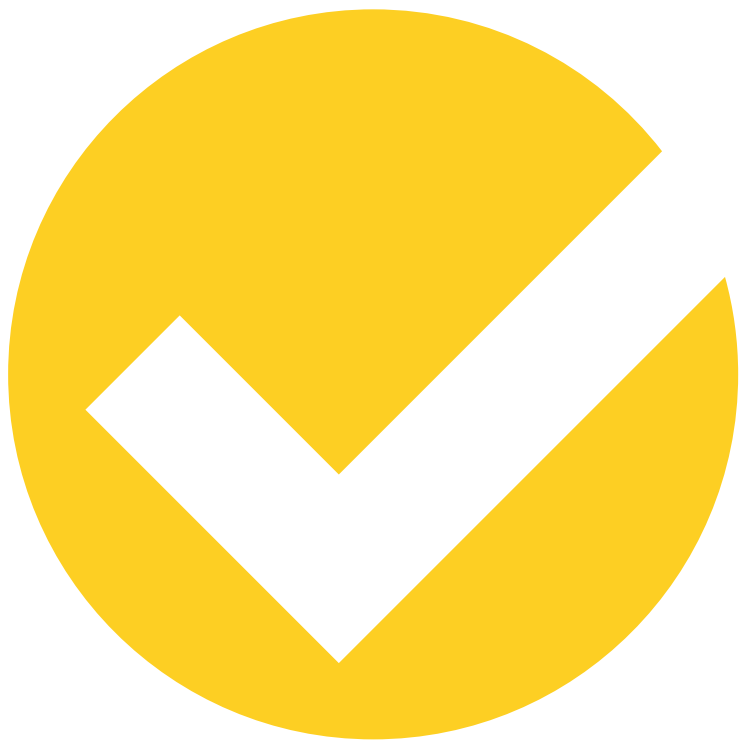

check for  
updates

Supplement: Supplementary file 1 [file micromachines-12-00358-s001.zip › Supplement/Definitions/logo-updates.pdf]

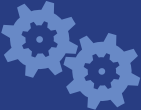

*micromachines*

Supplement: Supplementary file 1 [file micromachines-12-00358-s001.zip › Supplement/Definitions/micromachines-logo-eps-converted-to.pdf]

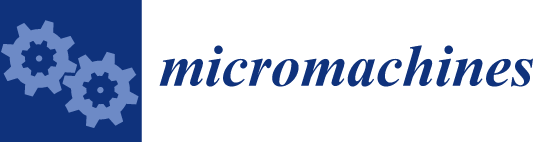

Supplement: Supplementary file 1 [file micromachines-12-00358-s001.zip › Supplement/Definitions/micromachines-logo.png]

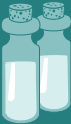

*molbank*

Supplement: Supplementary file 1 [file micromachines-12-00358-s001.zip › Supplement/Definitions/molbank-logo-eps-converted-to.pdf]

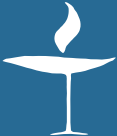

*religions*

Supplement: Supplementary file 1 [file micromachines-12-00358-s001.zip › Supplement/Definitions/religions-logo-eps-converted-to.pdf]

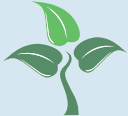

*sustainability*

Supplement: Supplementary file 1 [file micromachines-12-00358-s001.zip › Supplement/Definitions/sustainability-logo-eps-converted-to.pdf]

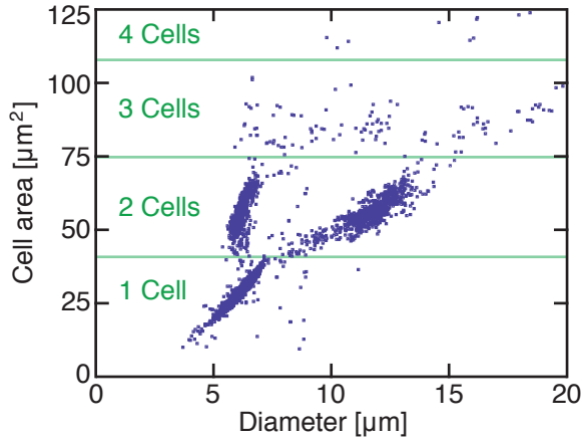

Supplement: Supplementary file 1 [file micromachines-12-00358-s001.zip › Supplement/Figures/FigS1_Helv.pdf]

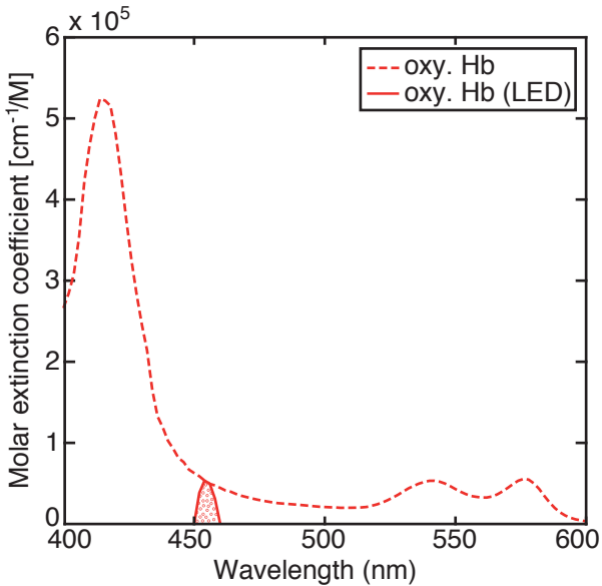

Supplement: Supplementary file 1 [file micromachines-12-00358-s001.zip › Supplement/Figures/FigS2_Helv.pdf]

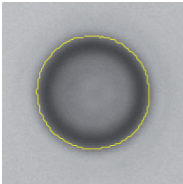

$z = -3 \mu\text{m}$

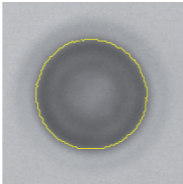

$z = 0 \mu\text{m}$

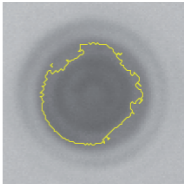

$z = +3 \mu\text{m}$

Supplement: Supplementary file 1 [file micromachines-12-00358-s001.zip › Supplement/Figures/FigS3_Helv.pdf]
